# Morphological data on soft ferromagnetic Fe_90_Ta_10_ thin films

**DOI:** 10.1016/j.dib.2019.104714

**Published:** 2019-10-25

**Authors:** Surabhi Shaji, Nikhil R. Mucha, Svitlana Fialkova, Dhananjay Kumar

**Affiliations:** Department of Mechanical Engineering, North Carolina Agricultural and Technical State University, Greensboro, NC, 27411, USA

**Keywords:** Thin films, Ferromagnetism, Atomic force microscopy

## Abstract

Iron-tantalum (Fe–Ta) thin films were synthesized on silicon (Si) (100) substrates using a pulsed laser deposition (PLD) technique. For the analysis of all reported data, please refer to our main article “Magnetic and electrical properties of Fe_90_Ta_10_ thin films [1]”. Morphological data confirm the amorphous nature of the film. Mesokurtic surface of the film was revealed using atomic force microscopy (AFM) analysis. The compositions of target and films were determined using x-ray fluorescence (XRF) data. The composition of Fe–Ta clusters, observed on the film surface, was measured using energy dispersive x-ray (EDX) analysis.

**Table:** Specifications Table:

Subject area	Physics
More specific subject area	Material Science
Type of data	AFM, SEM, EDX, XRF, Graphs
How data was acquired	Pulsed Laser Deposition - KrF Excimer laser from Coherent Compex Pro, Atomic Force Microscopy -NT-MDT NTEGRA Prima Modular scanning probe microscope. Scanning Electron Microscopy - Hitachi® SEM SU8000 X-ray Fluorescence Technique- Horiba XGT-7200 X-Ray Analytical Microscope Data analysis were done using Origin Pro software.
Data format	Raw and Analyzed
Experimental factors	Silicon substrates were cut to required dimensions using a diamond wheel. Vacuum was obtained overnight, and films were grown in the chamber using 20000 laser pulses with 10 Hz frequency and 380 mJ of energy.
Experimental features	Analysis were done using Hitachi field emission scanning electron microscope and NT-MDT NTEGRA Prima Modular scanning probe microscope configured with piezo sensor and high-resolution DLC coated tip.
Data source location	North Carolina Agricultural and Technical State University, Greensboro, North Carolina, USA
Data accessibility	All data is accessible within this article
Related Research Article	S. Shaji, N.R. Mucha, A.K. Majumdar, C. Binek, A. Kebede, D. Kumar, Magnetic and electrical properties of Fe90Ta10 thin films. Journal of Magnetism and Magnetic Materials https://doi.org/10.1016/j.jmmm.2019.165446

**Table:** 

**Value of the Data** • This data can be used to develop alloy thin films using PLD technique. • The data related to PLD parameters can be used to synthesize stoichiometric Fe–Ta thin films. • The variation in the deviation in the fitted data from the raw data could be used to discern different regimes of material's behavior.

## 1 Data

**Fig. 1:**
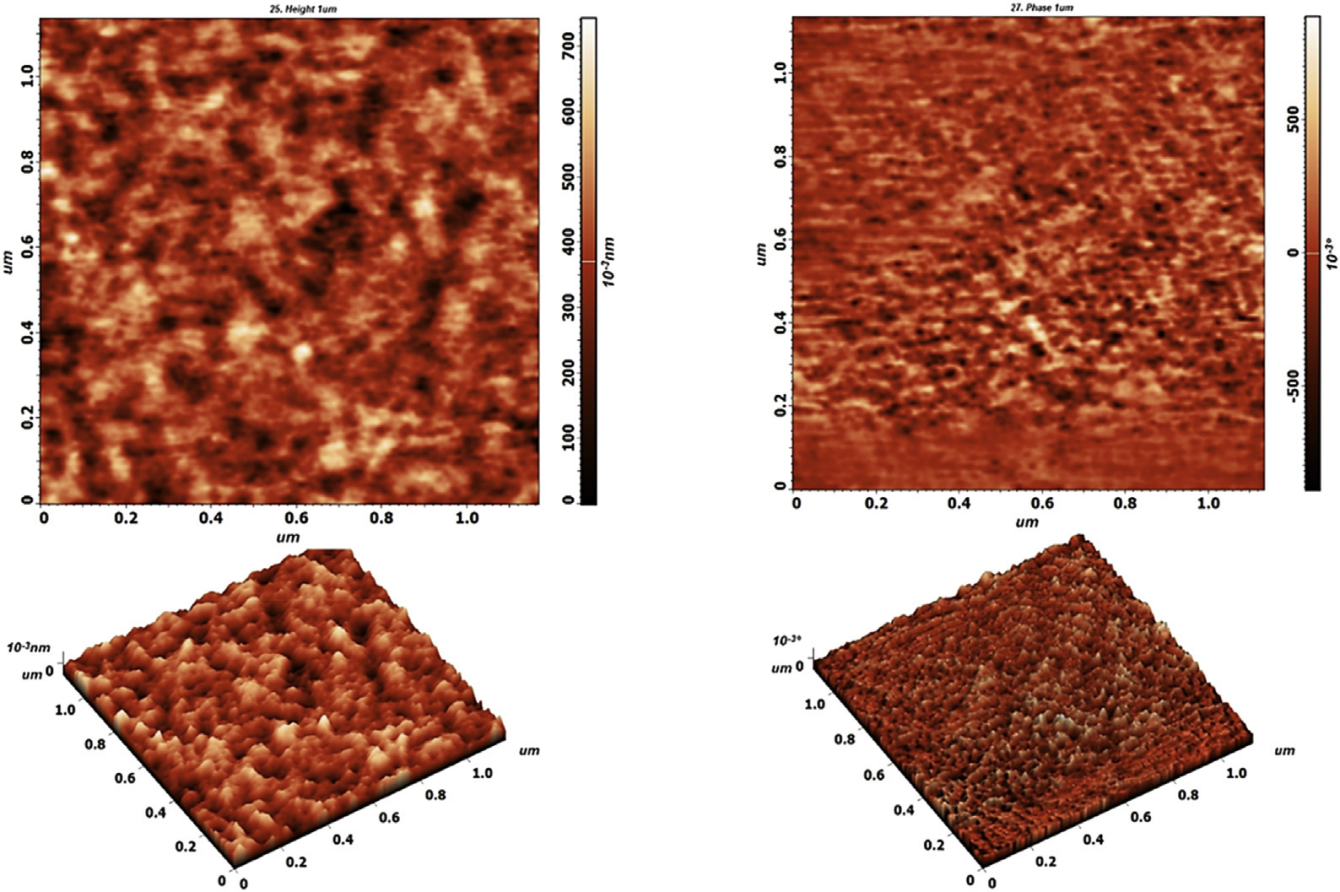
AFM analysis.

This data article reports the topography of Fe_90_Ta_10_ thin films, developed by pulsed laser deposition (PLD) technique at room temperature (RT). Fig. 1 shows the high-resolution AFM image data which is used to analyze the surface morphology of metallic films. The surface morphology of these films are explained using several other data obtained from AFM, such as roughness, height, amplitude etc. We have obtained an average roughness (R_ave_) of 0.0817 nm and root mean square roughness (R_rms_) of 0.125 nm on our samples from which height variations were calculated. Many peaks and valleys are observed in the images, which affects the R_ave_ and R_rms_ data values. It can be used to calculate the peak-to-valley difference [2,3]. The maximum peak height is obtained as 3.34 nm and maximum valley depth is 0.414 nm and therefore the calculated peak-to-valley height for this film is 3.75 nm. The shape parameters such as skewness (89.567) and kurtosis (5.227) data give an idea about the surface structure of the films like flatness and asymmetry. Here we have obtained high values for skewness which means the height distribution is uneven and there are more peaks than valleys [4]. Our kurtosis moment is greater than 3 indicating a Gaussian amplitude distribution. Thus, our sample surface can be called as Mesokurtic [5].

**Fig. 2:**
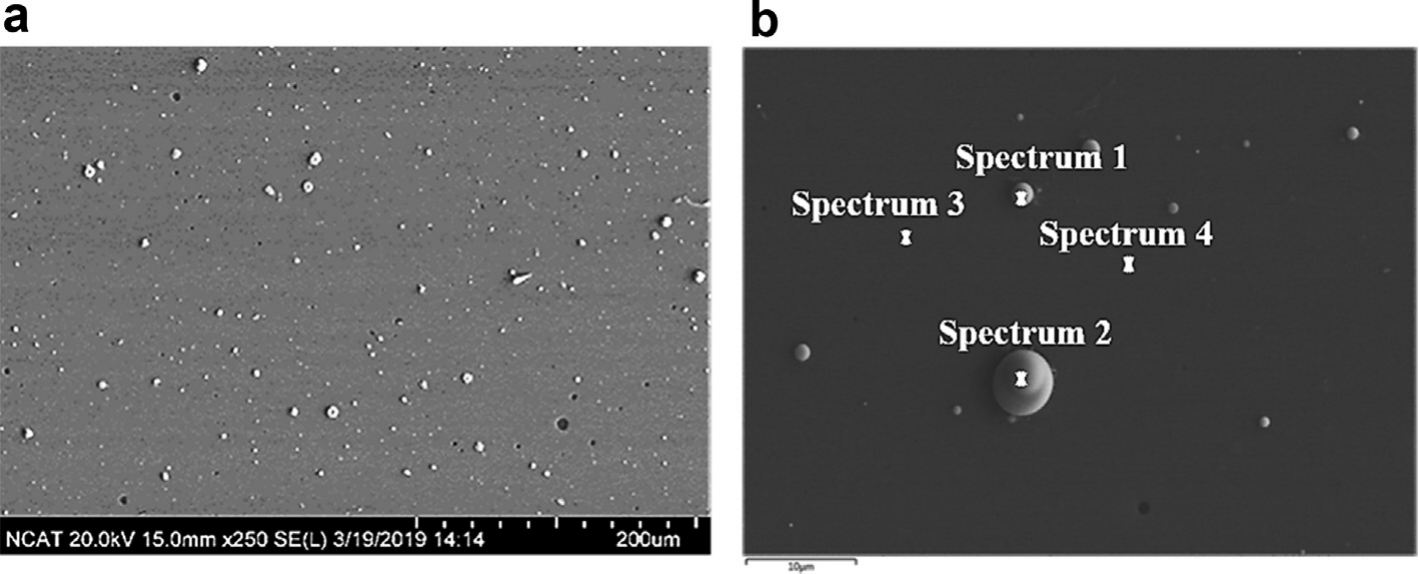
(a) Scanning Electron Microscopy images of Fe_90_Ta_10_ thin films at 200 μm scale (left) and (b) SEM images at 10 μm scale (right).

**Fig. 3:**
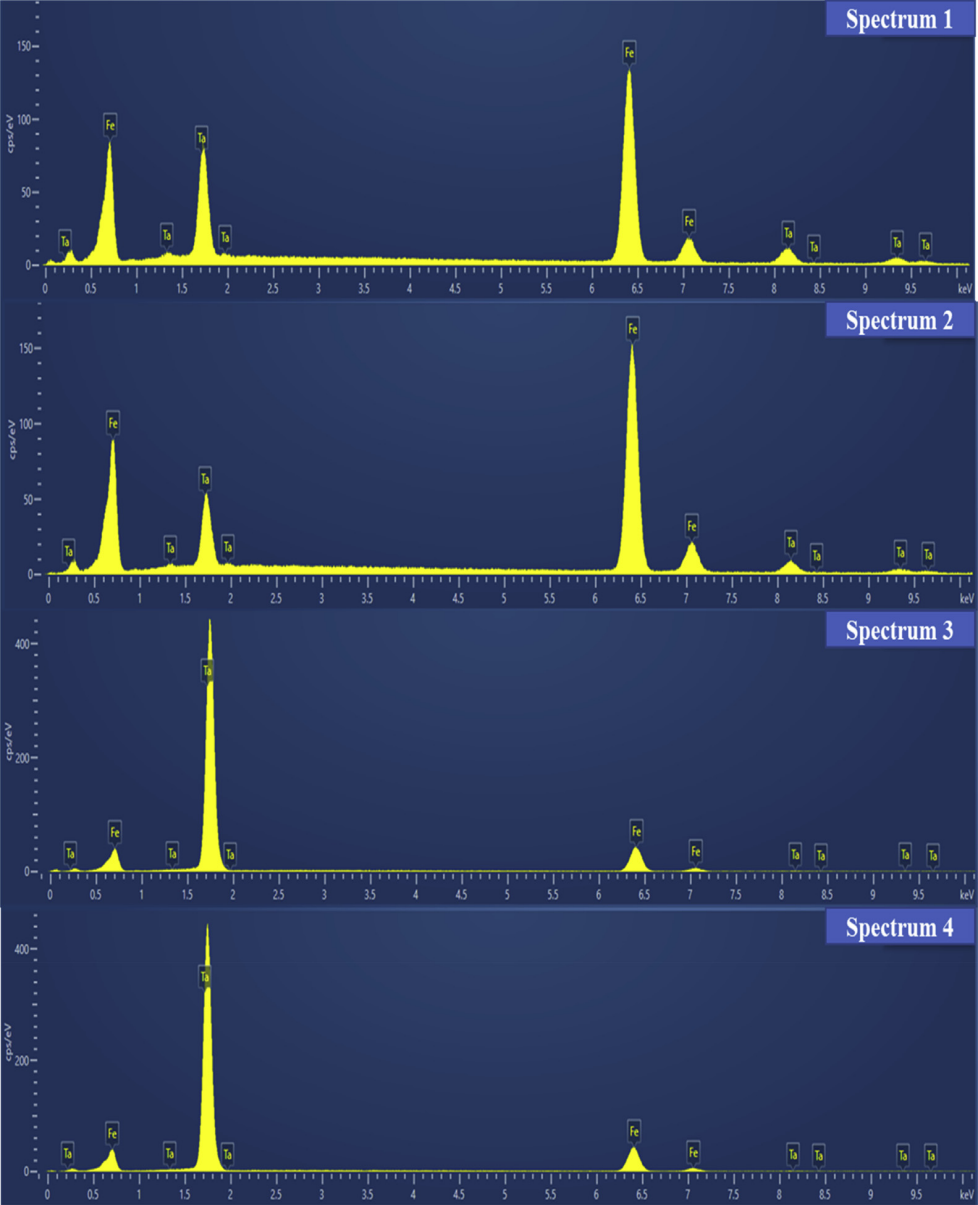
EDX analysis on Fe_90_Ta_10_ thin films.

Fig. 2 is the image data obtained from scanning electron microscope (SEM) for the room temperature (RT) deposited Fe_90_Ta_10_ thin films. A large number of white droplet shaped clusters are seen on the extremely smooth film surface which is evident from the SEM images. The absence of grains even at high magnification reveals the amorphous nature of the film which supports the XRD data reported in our main article [1]. EDX analysis was carried out on those droplets to confirm the material and composition by using the focused surface mapping technique and a point-by-point method. Four different sites were chosen as marked in Fig. 2(b) to analyze the composition. These data obtained from these four sites are plotted in spectrum 1 to 4 in Fig. 3. The statistical data obtained from EDX analysis for each spectrum is given in Table 1.

**Table 1:** Data obtained from EDX analysis of the four sites marked in Fig. 2(b).

Spectrum	Element	Line Type	Apparent Concentration	Intensity Correction	k Ratio	Wt.%	Wt.% Sigma	Atomic %
1	Fe	K series	225.95	1.04	2.25953	71.6	0.5	89.09
	Ta	L series	66.64	0.77	0.66636	28.4	0.5	10.91
2	Fe	K series	255.65	1.03	2.5565	80.25	0.48	92.94
	Ta	L series	46.1	0.75	0.46103	19.75	0.48	7.06
3	Fe	K series	72.25	1	0.72246	100	0	100
	Ta	L series	0	0.71	0	0	1.32	0
4	Fe	K series	72.47	1.01	0.72468	95.97	0.04	98.72
	Ta	L series	2.17	0.72	0.02168	4.03	1.24	1.28

The film composition also checked by XRF Analysis; the data obtained shows the composition is almost same as obtained in EDX analysis. The size of the sample used for topographical analysis was of 5 mm × 6 mm. Table 2 shows the composition of the film obtained using XRF analysis which is an average of 36 different sites on the film.

**Table 2:** Data obtained from XRF analysis.

Element	Line	Mass [%]	3sigma	Atomic [%]	Intensity[cps/mA]
Fe	K	92.01	2.27	97.39	3588.13
Ta	L	7.99	2.27	2.61	51.47

**Fig. 4:**
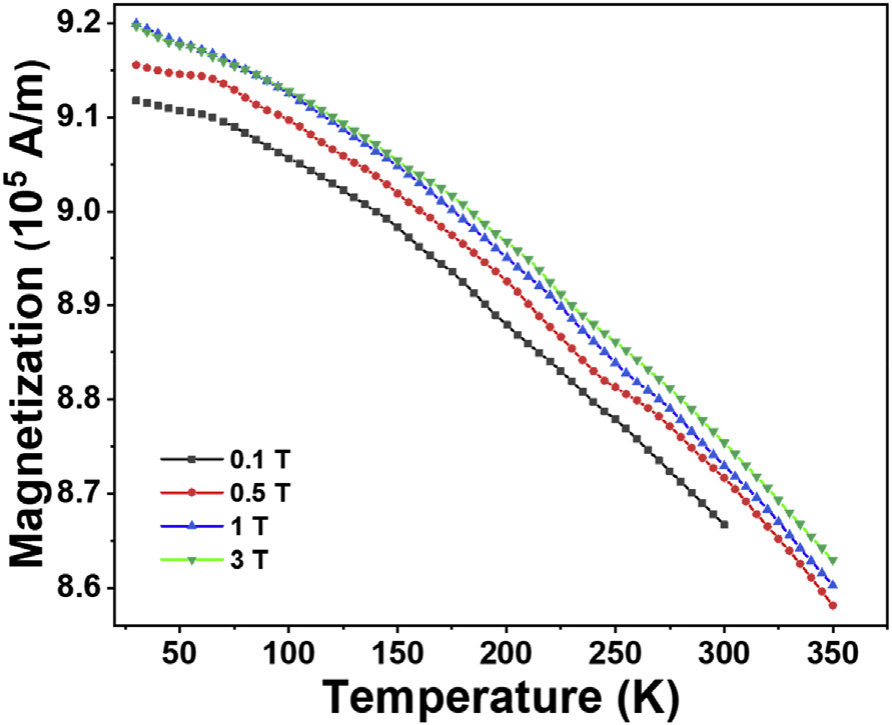
Temperature dependence magnetization plots for Fe_90_Ta _10_ thin films at various fields.

The temperature dependent magnetization at four fields (0.1T–3 T) is shown in Fig. 4 [1]. These magnetization (M) versus temperature (T) data were fitted to the following equation,

(1) $$\frac{M\left(T\right)-M\left(0\right)}{M\left(0\right)}=CT^{\frac{3}{2}}\;+\;DT^{\frac{5}{2}}$$

**Fig. 5:**
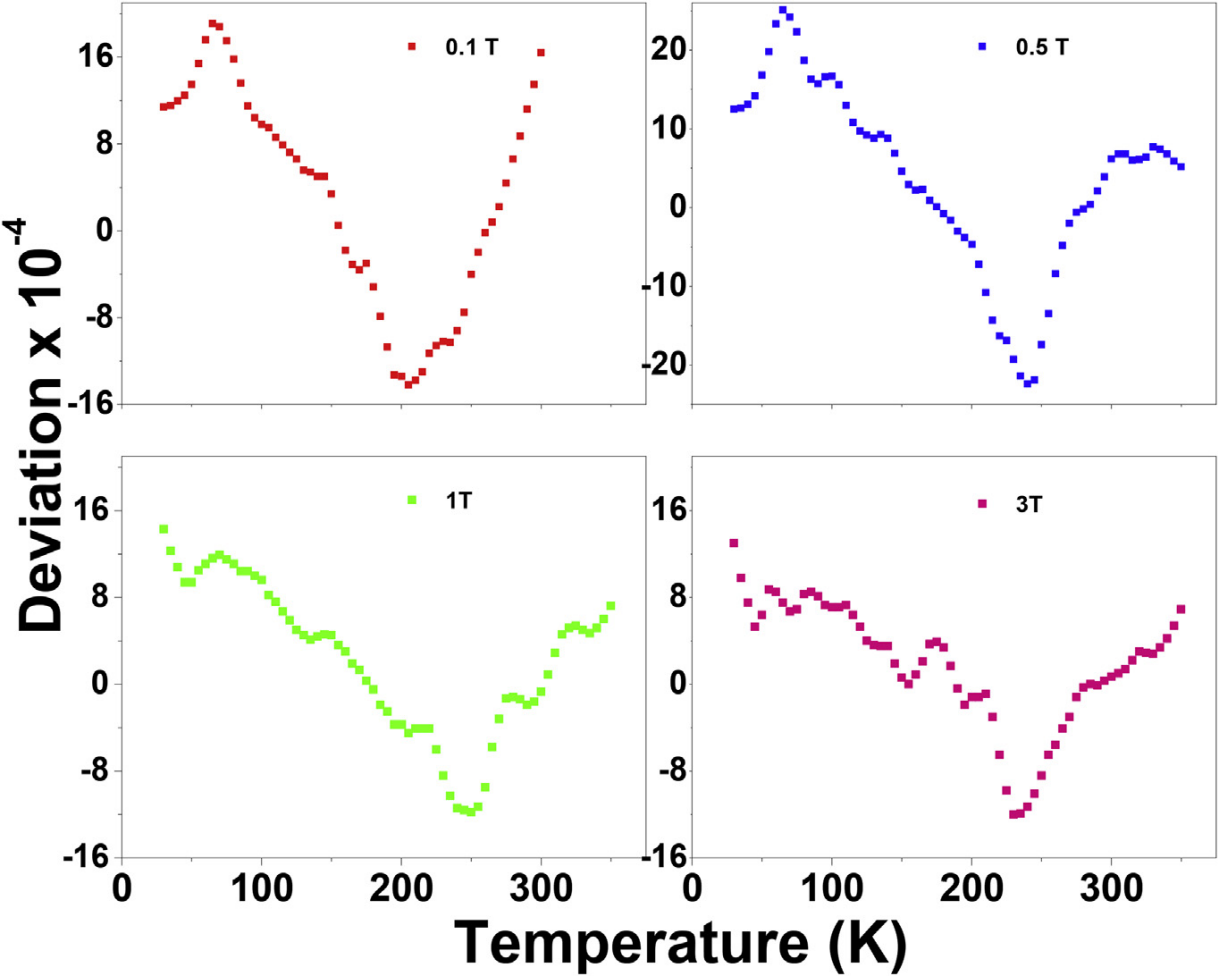
Deviation between raw data and best fitted data plotted over temperature.

The M-T fit data was used to find the deviation between the raw data and best fitted data for different fields and plotted over various temperatures as shown in Fig. 5. For all fields, same trend of variation with temperature is observed. The deviations in the order of 10^−4^ show the goodness of the fit. It was observed that the best fits of the data were obtained around mid-range temperatures for all fields.

## 2 Experimental design, materials and methods

### 2.1 Sample preparation

Silicon (100) wafers were cut into 10 mm × 10 mm size and cleaned using DI water, ethanol, acetone and methanol. The substrate was immersed in each of the alcohol solution and sonicated for 15 minutes using an ultrasonicator. The clean substrates were stored in a dry air desiccator until ready to be mounted in the PLD. To prevent oxidation, the samples were dipped in HF solution for 10 seconds just before mounting it on the stage in the PLD chamber.

### 2.2 Pulsed laser deposition technique (PLD)

**Fig. 6:**
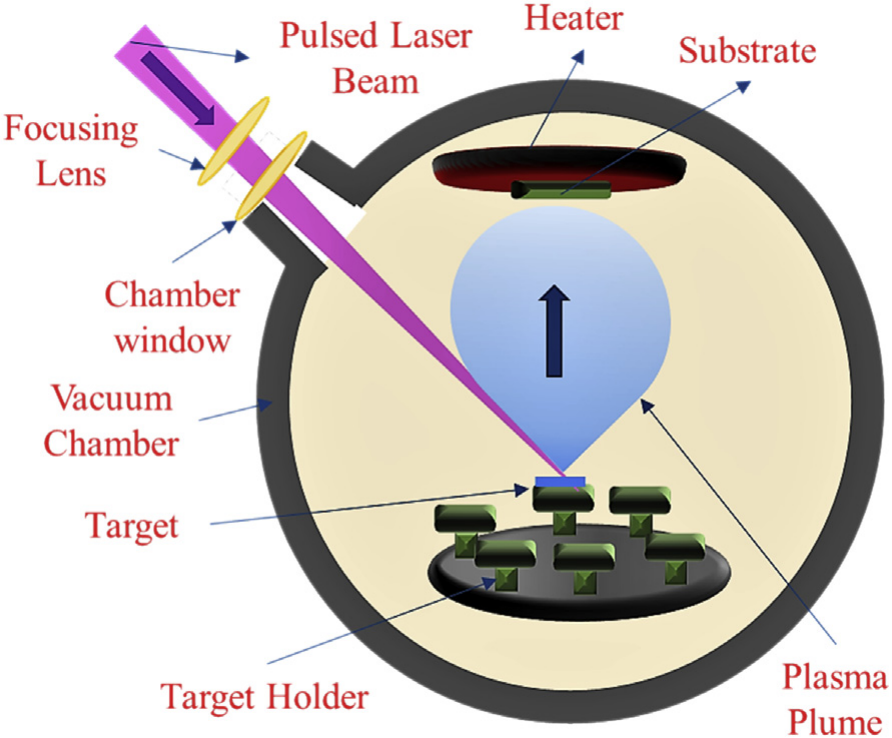
Schematic of pulsed laser deposition (PLD) technique.

PLD is a versatile technique used to fabricate a wide variety of thin films [[6], [7], [8]], yet there are various deposition parameters that need to be taken into account in order to fabricate good quality films. The schematic of a PLD process is shown in Fig. 6. In PLD, a pulsed laser beam with high energy strikes a target. The energy of laser is absorbed in the subsurface region of the solid target resulting in the melting and evaporation of the solid material [8,9]. This evaporation of target materials by the laser produces a highly luminous and transient plasma plume that expands rapidly away from the target surface as shown in Fig. 6. This plasma plume of the vaporized material contains neutrals, ions, electrons etc.

The ablated material is collected on a substrate placed opposite to the target where it condenses and leads to the growth of thin films [8,9]. Variables like laser fluence, background gas pressure and substrate temperature affects the properties of the film. These properties can be manipulated by controlling the variables to suit the desired applications. In our PLD experiments, we have used krypton fluoride (KrF) excimer laser source (Coherent Compex Pro). The wavelength of KrF was 248 nm. The substrate to target distance was maintained between 4.5 and 5 cm. The laser was operated at a pulse rate of 10 Hz with energy of 380 mJ/pulse and the number of pulses was set to 20000 pulses for all depositions. Films were deposited at various substrate temperatures ranging from room temperature (RT) to 700 °C. The temperature difference at the surface of the substrate causes an increase in the surface mobility and energy of the condensing species during nucleation and growth of the film [10]. The film was grown in a vacuum of the order of 10^−7^ Torr.

### 2.3 Atomic force microscopy

The surface morphology was analyzed using a NT-MDT NTEGRA Prima Modular scanning probe microscope. Scanning with piezo-sensor (range 100 × 100 μm), and high-resolution DLC-coated tip (NT-MDT, model no. HA_HR_DLC, R = 5–6 nm) high resolution measurements allows the study of surface morphology of metallic films along with the material properties and compositional mapping of the sample surfaces. The thorough knowledge of the approaches in measurements and data analysis using AFM technique are important for the correct interpretation of topographic features of the surfaces. The accurate surface properties obtained are mainly influenced by the scan area, resolution and image data analysis procedures [4].

### 2.4 Scanning Electron Microscopy – EDX

Scanning electron microscope (SEM) is the most widely employed thin film and coating characterization instrument. Hitachi® SEM SU8000 with an EDX attachment was used to visualize the Fe_90_Ta_10_ thin films with 20 mA current 50 kV voltage. Composition of the film and cluster formation on the surface was confirmed using mapping and point-by-point EDX analysis.

### 2.5 X-ray fluorescence (XRF)

The XRF technique acts as a merger between optical observations and elemental analysis functions and mainly used to determine the elemental composition of materials. The secondary x-rays emitted from a sample, which is excited by a primary x-ray source, is captured and analyzed to determine the presence of elements on the sample. These secondary x-rays emitted by each elements on the sample are unique like a fingerprint, which is why the XRF technology is excellent to determine the material composition. We have used Horiba XGT – 7200 X-ray Analytical Microscope to confirm the elemental composition of both target and film.
